# Effects of 17β-oestradiol and norethisterone acetate on sulfonation and sialylation of gonadotrophins in post-menopausal women

**DOI:** 10.3109/03009730903573253

**Published:** 2010-04-07

**Authors:** Leif Wide, Tord Naessén, Karin Eriksson

**Affiliations:** ^1^Department of Medical Sciences, Clinical Chemistry, University Hospital, UppsalaSweden; ^2^Department of Women's and Children's Health, Obstetrics and Gynaecology, University Hospital, UppsalaSweden

**Keywords:** FSH isoforms, LH isoforms, post-menopause, sialic acid, sulfonated N-acetylgalactosamine

## Abstract

**Background:**

The number of terminal sialic acid and sulfonated N-acetylgalactosamine (SO_3_-GalNAc) on gonadotrophins in serum varies during the menstrual cycle and changes at menopause, suggesting that gonadal steroids modify their oligosaccharide synthesis. Our objective was to determine the effects of 17β-oestradiol (E_2_) and a progestogen, norethisterone acetate (NETA), on the sulfonation and sialylation of gonadotrophins in post-menopausal women.

**Methods:**

Serum samples were obtained from eight post-menopausal women treated with 20 mg E_2_ implants every 6 months, from four women who in addition were treated daily with 5 mg NETA orally for a 2-week period, and from four women who got this NETA treatment during a 4-week period. Sera from 11 non-treated post-menopausal women served as a reference group. The gonadotrophin serum concentrations, the number of SO_3_-GalNAc and sialic acid residues per serum luteinizing hormone (LH) and follicle-stimulating hormone (FSH) molecule, and the distributions of molecules with 0-1-2-3-4 sulfonated residues were measured.

**Results:**

The E_2_-treated post-menopausal women had considerably less (*P* < 0.001) sialic acid and slightly more (*P* < 0.01) SO_3_-GalNAc per serum LH and FSH molecule than the non-treated. Two weeks of NETA treatment increased the sulfonation of LH (*P* < 0.01) and FSH (*P* < 0.05) concomitantly with decreased (*P* < 0.05) sialylation of LH.

**Conclusion:**

The primary effect of E_2_ treatment was a decrease in sialylation and, due to competition for the same substrate, a secondary and consequentially minor increase in sulfonation of LH and FSH. The primary effect of the NETA therapy was an increase in the sulfonation of LH and FSH concomitantly with secondary and consequentially decreases in sialylation of LH.

## Introduction

Human luteinizing hormone (LH) and follicle-stimulating hormone ( FSH) are synthesized in the pituitary and circulate in blood as individual spectra of large numbers of different isoforms (1,2). The asparagine-bound oligosaccharides, three on LH and four on FSH, vary between isoforms with respect to the number of terminal sialic acid and sulfonated N-acetylgalactosamine (SO_3_-GalNAc) residues per molecule (2,3). The composition of such LH and FSH isoforms in serum varies during the menstrual cycle, changes after menopause, and is different in women with polycystic ovarian syndrome and in healthy men compared with healthy women (2). These results suggested that gonadal steroids, such as oestrogen, progesterone, and androgen, modify the enzyme activity in the sialylation and/or the sulfonation pathways of the gonadotrophin synthesis in the human pituitary.

The effect of gonadal steroids on gonadotrophin glycosylation was extensively studied in animal experiments in which LH and FSH were extracted from the pituitaries (4). It was concluded that oestradiol reduces while testosterone increases the sialylation. In the human, similar studies have been restricted to analyses of FSH and LH in blood. Effects of gonadal steroids on the synthesis of human gonadotrophin isoforms have been observed as changes in net charge of the molecules (5–15). Both the sialic acid and the sulfonated GalNAc residues are negatively charged and determine the anionic character of the oligosaccharides and thereby the net charge of the gonadotrophin molecules. These two negatively charged residues have biological implications, as the number of sialic acid and sulfonated GalNAc residues on human LH and FSH determines their clearance from the circulation. More sulfonated GalNAc per molecule decreases and more sialic acid per molecule increases the half-life of human LH and FSH in blood (16,17).

Administration of 17β-oestradiol (E_2_) implants in post-menopausal women was shown to counteract the formation of the more anionic (acidic) forms of both LH and FSH normally secreted in these women (8). The effect of a progestogen, norethisterone acetate, on the net charge of the isoforms in the E_2_-treated women was significant and time-related (11). More anionic forms appeared in the circulation during the first 2 weeks of progestogen treatment, while during the following 2 weeks the isoforms became more cationic (basic) again. The objective of the present study was to determine to what extent these effects of E_2_ and norethisterone acetate (NETA) on the molecular net charge were due to changes in sialic acid and/or sulfonated GalNAc residues on the gonadotrophins. The average number of sialic acid and sulfonated GalNAc residues per molecule of LH and FSH in serum was estimated with a method based on neuraminidase treatment, electrophoresis, and immunofluorometric hormone assays (2). In addition, the distributions of isoforms with zero to four SO_3_-GalNAc residues per molecule were measured. The results were compared with those of 11 non-treated post-menopausal women.

## Material and methods

### Subjects, serum samples, and experimental design

Serum samples were analysed from eight women, mean age 65 years (range 50–79), treated with 20 mg E_2_ implanted subcutaneous in pellets (Organon Laboratories Ltd, UK) every 6 months (group: E_2_-implant). The samples were obtained 4–18 (mean 15.6) weeks after the insertion of the E_2_ implant, and the mean E_2_ level was 352 pmol/L (range 270–420). Serum samples were also analysed from four women, mean age 64 years (range 50–79), who in addition to this E_2_ therapy for a mean of 11 weeks (range 6–20) were treated for 2 weeks with oral therapy of 5 mg NETA (Primolut-Nor, Scherman AG, Germany) daily (group: E_2_+2wNETA). Their mean E_2_ level was 286 pmol/L (range 230–320). Serum samples were also analysed from four women after 4 weeks of the NETA treatment (group: E_2_+4wNETA), mean age 58 years (range 50–69), with a mean of 13 weeks (range 8–22) after insertion of the E_2_ implant. Their mean E_2_ level was 338 pmol/L (range 310–370). Two patients, A and B, participated in all three treatment groups. Patient A was treated twice with NETA, at 4 and at 18 weeks after insertion of the E_2_ implant, and serum samples were obtained before and after 2 and 4 weeks of NETA treatment. All the women had been hysterectomized and experienced menopausal symptoms before the first E_2_ implant, and they had been treated for a mean of 12 years (range 1.5–21). Serum specimens from 11 non-treated post-menopausal women, mean age 65 years (range 61–77), constituted a reference group. No individual had the common variant form of LH (17–19). The study was approved by the local Ethics Committee.

### Analytical methods

The study comprises a total of 3,000 gonadotrophin assays on the 30 sera and includes neuraminidase treatment and two electrophoreses of all sera and analyses to exclude the occurrence of the common genetic variant form of LH (17–19) among the patients. The concentrations of LH and FSH in serum samples and in 200 μL of fractions eluted after 0.10% agarose suspension electrophoresis were measured using sandwich fluoroimmunoassays (Delfia, PerkinElmer-Wallac Oy, Turku, Finland), as previously described (20). Gonadotrophin values were expressed in IU/L using the International Standards for pituitary LH (80/552) and FSH (94/632) as reference standards. The detection limits in serum were 0.02 IU/L, and the interassay coefficient of variation (CV) was less than 3% for both hormones.

The average number of sialic acid and sulfonated GalNAc residues per LH and FSH molecule and the distribution of molecules with zero to four sulfonated GalNAc were estimated, as previously described (2). The method is based upon the observation by Green and Baenziger (3) that these two negatively charged residues determine the anionic character of N-linked oligosaccharides on the gonadotrophins. All serum samples were analysed before and after neuraminidase treatment with an electrophoretic technique using a 0.10% agarose suspension in veronal buffer at pH 8.7 (2,21).

### Statistical analyses

Results are presented as mean values ± SEM. The mean values of the two serum samples of patient A in each treatment group were used in the statistical analyses. Statistical comparisons were made with non-parametric Mann-Whitney test. Relationships were identified by calculating Spearman's *r_s_*. A *P*-value < 0.05 was considered to be significantly different.

## Results

### Effects of E_2_ implants

The serum concentrations of FSH and LH and the number of sialic acid and sulfonated GalNAc residues per molecule in the non-treated and the E_2_-implant groups are given in Table I. The gonadotrophin serum levels of the E_2_-implant group were significantly lower (*P* < 0.01 for LH and < 0.001 for FSH) than in the reference group of non-treated post-menopausal women. The number of sialic acid residues per molecule was significantly (*P* < 0.001) lower in the E_2_-implant than in the non-treated group of both LH and FSH. The corresponding number of sulfonated GalNAc residues was increased (*P* < 0.01) on both gonadotrophins in the E_2_-implant group.

**Table I.** Sialic acid and sulfonated GalNAc residues per LH and FSH molecule in serum and frequency of isoforms with zero to four sulfonated GalNAc residues. Four groups of post-menopausal women: non-treated, treated with 20 mg E_2_ implant, and, in addition to E_2_, with 2 and 4 weeks of oral therapy with 5 mg NETA daily.

Group	Non-treated	E_2_-implant	E_2_+2wNETA	E_2_+4wNETA
Number of women	11	8	4	4
LH; mean ± SEM				
Serum level (IU/L)	24.4 ± 1.80	10.2 ± 2.21^b^	3.08 ± 0.56^a^	1.48 ± 0.48^a,d^
Sialic acid residues	2.64 ± 0.04	2.14 ± 0.04^c^	1.86 ± 0.04^a^	1.57 ± 0.06^a,d^
Sulfonated GalNAc	0.99 ± 0.04	1.15 ± 0.03^b^	1.64 ± 0.03^b^	1.80 ± 0.03^a,d^
Ratio Sial./Sulf.	2.70 ± 0.11	1.87 ± 0.07^c^	1.14 ± 0.04^b^	0.87 ± 0.04^a,d^
Negatively charged residues	3.64 ± 0.03	3.29 ± 0.03^c^	3.50 ± 0.02^b^	3.37 ± 0.05
Zero SO_3_-GalNAc (%)	30.7 ± 2.44	28.1 ± 0.90	14.2 ± 2.62^b^	6.04 ± 2.43^d^
One SO_3_-GalNAc (%)	46.9 ± 3.54	40.3 ± 2.08	30.8 ± 2.91	28.6 ± 2.96
Two SO_3_-GalNAc (%)	15.9 ± 2.57	21.5 ± 2.40	37.5 ± 2.26^b^	45.5 ± 11.2^d^
Three SO_3_-GalNAc (%)	5.80 ± 1.40	8.68 ± 2.00	14.4 ± 1.65	18.9 ± 6.77
Four SO_3_-GalNAc (%)	0.79 ± 0.36	1.42 ± 0.65	3.82 ± 1.15	0.91 ± 0.77
Two to four SO_3_-GalNAc (%)	22.4 ± 1.92	31.6 ± 1.89	55.7 ± 1.51^b^	65.3 ± 4.58^a,d^
FSH; mean ± SEM				
Serum level (IU/L)	56.5 ± 4.01	10.4 ± 2.63^c^	4.20 ± 1.31	2.21 ± 0.41^a,d^
Sialic acid residues	7.55 ± 0.03	6.53 ± 0.11^c^	6.68 ± 0.07	6.17 ± 0.10^a^
Sulfonated GalNAc	0.22 ± 0.03	0.39 ± 0.03^b^	0.58 ± 0.04^a^	0.80 ± 2.73^a,d^
Ratio Sial./Sulf.	44.5 ± 9.98	17.4 ± 1.49^c^	11.7 ± 1.0	7.71 ± 0.39^a,d^
Negatively charged residues	7.55 ± 0.03	6.92 ± 0.12^c^	7.26 ± 0.05^a^	7.00 ± 0.06^a^
Zero SO_3_-GalNAc (%)	86.6 ± 2.08	74.1 ± 2.34^b^	63.6 ± 2.90^a^	48.6 ± 2.46^a,d^
One SO_3_-GalNAc (%)	4.93 ± 1.17	13.2 ± 2.49^b^	15.1 ± 1.39	24.0 ± 3.31
Two SO_3_-GalNAc (%)	8.07 ± 1.03	12.1 ± 1.32^a^	20.8 ± 2.11^b^	26.3 ± 1.15^a,d^
Two to four SO_3_-GalNAc (%)	8.45 ± 1.00	12.8 ± 1.14^a^	21.3 ± 1.63^b^	27.4 ± 1.62^a,d^

Statistical comparisons with non-parametric Mann-Whitney test. ^a^*P* < 0.05 versus preceding group. ^b^*P* < 0.01 versus preceding group. ^c^*P* < 0.001 versus preceding group. ^d^*P* < 0.01 versus E_2_-implant group.

The decrease in sialic acid was larger than the increase in sulfonated residues resulting in decreased (*P* < 0.001) total number of negatively charged residues per molecule on both LH (minus 0.34 ± 0.04) and FSH (minus 0.63 ± 0.11) in the E_2_-implant group compared with the non-treated group of women. The ratio of sialic acid to sulfonated GalNAc residues per molecule was lower (*P* < 0.001) in the E_2_-implant group than in the non-treated women: for LH 1.9 versus 2.7, and for FSH 17 versus 44 (Table I).

### Effects of NETA in three individual studies

**Figure 1.**
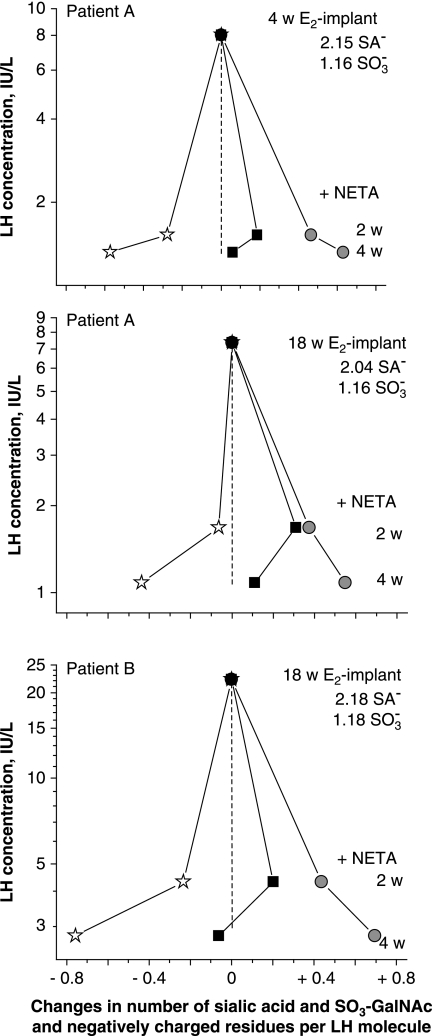
Relationship between LH concentration and changes in the number of sialic acid (open star) and sulfonated (filled circle) residues and negatively charged residues (filled square) on the oligosaccharides per LH molecule after 2 and 4 weeks of NETA therapy in two E_2_-implant-treated post-menopausal women. The average numbers of sialic acid (SA^-^) and sulfonated (SO_3_^-^) residues per serum LH molecule at the start of NETA treatment are given (w = weeks).

**Figure 2.**
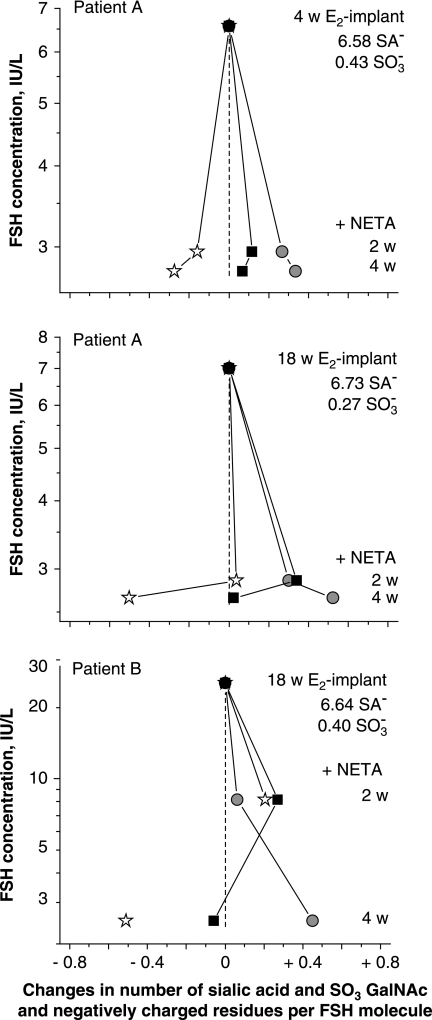
Relationship between FSH concentration and changes in the number of sialic acid (open star) and sulfonated (filled circle) residues and negatively charged residues (filled square) on the oligosaccharides per FSH molecule after 2 and 4 weeks of NETA therapy in two E_2_-implant-treated post-menopausal women. The average numbers of sialic acid (SA^-^) and sulfonated (SO_3_^-^) residues per serum FSH molecule at the start of NETA treatment are given (w = weeks).

Sera from three individual studies with samples taken before and after 2 and 4 weeks of NETA treatment were analysed. Two of these were from one patient A, starting the NETA treatment at weeks 4 and 18 after insertion of the E_2_ implant, and the third from patient B with start of NETA treatment at E_2_ implant week 18. The changes in sialic acid and sulfonated residues and in total number of negatively charged residues are shown for LH in Figure 1 and for FSH in Figure 2.

There was in all three cases an increase in the number of sulfonated residues on LH after 2 weeks of treatment which was larger than the decrease in sialic acid residues. Therefore, the net effect was an increase in the total number of negatively charged residues. During the following 2 weeks of treatment the number of sulfonated residues further increased. During this period there was a more dramatic decrease in sialic acid residues. This decrease was larger than the increase in sulfonated residues resulting in a change of the total number of negatively charged residues back to values close to those before treatment with NETA.

FSH showed a similar change in sulfonated residues but less pronounced compared to LH. The changes in sialic acid during 2 weeks of NETA therapy varied from a slight decrease to a slight increase, and the net charge became more negative in all three cases. During the next 2 weeks of NETA treatment the contents of sialic acid on FSH decreased more than the number of sulfonated residues increased. The net effect was a decrease in negatively charged groups, and the charge was similar to that at the start of treatment.

### Effects of NETA treatment—groups compared

The serum concentrations of LH and FSH and the number of sialic acid and sulfonated GalNAc residues per molecule in the E_2_-implant, E_2_+2wNETA, and E_2_+4wNETA groups are given in Table I. The serum gonadotrophin concentrations decreased significantly (*P* < 0.01) after 4 weeks of NETA treatment. The mean levels of LH and FSH were suppressed to 6% and 4%, respectively, of those of the non-treated post-menopausal women.

**Figure 3.**
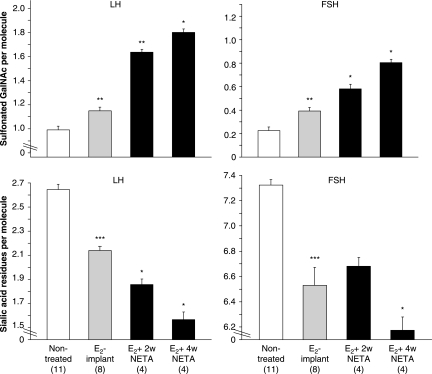
Mean ± SEM of average number of sialic acid (lower panels) and sulfonated GalNAc (upper panels) residues per serum LH (left panels) and FSH (right panels) molecule in four groups of post-menopausal women. NETA was given to E_2_-implant-treated women, and sera were taken at the start (E_2_-implant) and after 2 (E_2_+2wNETA) and 4 (E_2_+4wNETA) weeks of treatment (non-treated = a reference group of non-treated post-menopausal women). Statistical comparison with preceding group. *P < 0.05; **P < 0.01; ***P < 0.001. Figures in parentheses indicate number of women in each group.

The change in sialic acid and sulfonated GalNAc residues per LH and FSH molecule after 2 and 4 weeks of NETA treatment is illustrated in Figure 3. The number of sulfonated residues increased for both gonadotrophins—more pronounced for LH than for FSH. The number of sialic acid residues on LH decreased during the first 2 weeks of NETA treatment and continued to decrease during the second 2 weeks of treatment. The number of sialic acid residues per FSH molecule was unchanged after 2 weeks of NETA treatment and decreased during the second 2 weeks of treatment. The net effect of the first 2 weeks of treatment with NETA was an increase in the total number of negatively charged residues per molecule which was 0.20 ± 0.05 (*P* < 0.01) for LH and 0.34 ± 0.17 (*P* < 0.05) for FSH. During the last 2 weeks of NETA treatment the total number of negatively charged residues decreased with 0.13 ± 0.05 (*P* = 0.05) on LH and with 0.28 ± 0.09 (*P* < 0.05) on FSH.

The ratios of sialic acid to sulfonated residues on LH and FSH during the NETA treatment are given in Table I. The ratios decreased significantly (*P* < 0.01) after 4 weeks to 0.87 for LH and to 7.71 for FSH.

### Sialic acid and SO_3_-GalNAc residues per molecule versus serum levels of LH and FSH

**Figure 4.**
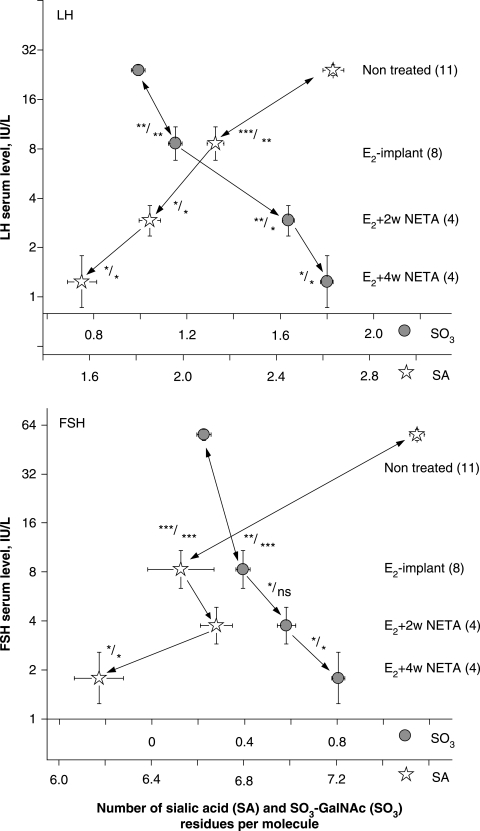
Relationship between serum LH (upper panel) and FSH (lower panel) concentration and number of sialic acid (open star) and sulfonated GalNAc (filled circle) residues per molecule in four groups of post-menopausal women. NETA was given to E_2_-implant-treated women, and sera were taken at the start (E_2_-implant) and after 2 (E_2_+2wNETA) and 4 (E_2_+4wNETA) weeks of treatment (non-treated = a reference group of nine non-treated post-menopausal women). Significance of difference in number of residues/concentration is indicated. *P < 0.05; **P < 0.01; ***P < 0.001; ns = not significant. Figures in parentheses indicate number of women in each group.

The relationships between the degrees of sialylation and sulfonation of the gonadotrophins and their serum levels are shown in Figure 4. The figure illustrates that the relationships between the number of sulfonated GalNAc per molecule and the serum levels of LH and FSH are negative and that the corresponding relationships for sialic acid are positive. The estimated coefficients of correlation (Spearman's *r_s_*) were for LH concentration versus sulfonated GalNAc residues −0.73 and versus sialic acid residues 0.86, and the corresponding values for FSH were −0.82 and 0.83, respectively (*P* < 0.0001 for all *r_s_*-values; *n* = 27).

### Distributions of LH and FSH molecules with different number of SO_3_-GalNAc residues

The distributions of LH and FSH isoforms with different number of sulfonated GalNAc residues per molecule in serum are given as mean values in per cent of total in Table I. The distributions of LH isoforms were similar for the non-treated and the E_2_-implant-treated groups. During the NETA treatment there was a decrease (*P* < 0.01) of the non-sulfonated LH isoforms from 28% to 6% and an increase (*P* < 0.01) of the isoforms with two to four sulfonated GalNAc residues from 32% to 65%.

The frequency of non-sulfonated isoforms of FSH decreased (*P* < 0.01) from 87% in the non-treated to 74% in the E_2_-implant group. There was then a further decrease (*P* < 0.01) to 49% after 4 weeks of the NETA treatment. The frequency of FSH isoforms with two to four sulfonated residues increased (*P* < 0.01) from 8.4% in the non-treated to 27% in the E_2_+4wNETA group.

## Discussion

In this study we have examined the effect of oestradiol and of the addition of a progestogen, NETA, on the number of sialic acid and sulfonated GalNAc residues on serum LH and FSH in post-menopausal women. Our results indicate that both the oestradiol and the progestogen modulate the sulfonation and the sialylation of the oligosaccharides and suggest that oestradiol predominantly inhibits the sialylation pathway while the main effect of the progestogen is an enhancement of the sulfonation pathway.

The biochemical events leading to terminal sialylation or sulfonation of LH have been reviewed by Baenziger (22). The sulfonation pathway leading to terminal SO_3_‐4GalNAc is first regulated by a peptide-specific β1-4GalNAc-transferase adding GalNAc to the subterminal GlcNAc residue on the biantennary glycan chains. This β1-4 linked GalNAc moiety is then sulfonated by a sulfotransferase. This sulfonation pathway occurs in competition with a β1-4galactosyltransferase adding galactose to the same subterminal GlcNAc in a sialylation pathway leading to terminal sialic acid. A Pro-Leu-Arg tripeptide motif on the β-subunit of LH and a cluster of cationic amino acids (Pro-Leu-Arg-Ser-Lys-Lys) within an α-helix on the α-subunit are recognized by the peptide-specific β1-4GalNAc-transferase leading to a considerably increased rate of GalNAc transfer to the LH molecule (23,24). The tripeptide motif on β-LH is not present on the FSH β-subunit, and the α-subunit recognition motif is thought to be masked by the β-subunit of FSH. Therefore, the activity of the peptide-specific β1-4GalNAc-transferase is low, and the sialylation pathway dominates on FSH (22).

The E_2_-treated post-menopausal women had considerably less (*P* < 0.001) sialylated and slightly more sulfonated isoforms than the non-treated. This is interpreted as an inhibition of the sialylation pathway with the consequence that more of the substrate, the subterminal GlcNAc, is available for the sulfonation pathway. This seems to be a more likely explanation to the slightly higher levels of sulfonated GalNAc in the E_2_-implant group than the alternative: a direct enhancement of the sulfonation pathway.

Our observations on the effects of oestradiol are in agreement with those of two animal studies in which the oestradiol effects on the enzymes involved in the glycosylation of the oligosaccharides were investigated (25,26). The effect of oestrogen on one of the pituitary enzymes in the sialylation pathway, that adds sialic acid to galactose, was investigated by Damián-Matsumura et al. (26) in the female rat. Administration of oestradiol benzoate to castrated female rats was shown to significantly reduce the mRNA level for the pituitary α2,3-sialyltranferase. Dharmesh and Baenziger (25) studied the effect of oestrogen on the enzymes in the sulfonation pathway. They showed that in the female rat the levels of GalNAc-transferase and sulfotransferase in the pituitary were regulated by oestrogen in a similar manner as their substrate, the LH molecules. The proportion of oligosaccharides terminating with sulfonated GalNAc remained constant.

During the first 2 weeks of the progestogen therapy the sulfonation of both LH and FSH increased. The total number of negatively charged residues increased significantly during this period. We interpret the effect of the progestogen as an enhancement of the enzyme activities along the sulfonation pathway. A consequence of an increased activity in the sulfonation pathway during the progestogen therapy is a decrease in available subterminal GlcNAc, the substrate for the galactosyltransferase, for the sialylation pathway. This is a likely explanation to the decreased sialylation of LH during the first 2 weeks of NETA therapy.

The remarkable time-related change in the net charge of the gonadotrophins between 2 and 4 weeks of NETA therapy, as previously reported (11), can now be explained by the findings of the present study. During this prolonged period of NETA therapy, the number of sialic acid residues per molecule of LH and FSH decreased more than the number of sulfonated residues increased, leading to a decrease in negatively charged residues on the hormones. We suggest two possible explanations to this ‘oestrogen-like’ effect during the last 2 weeks of NETA treatment. A decreased sex hormone binding globulin (SHBG) level induced by the NETA therapy is expected to lead to more of free E_2_ in the circulation (11,27). An alternative possible explanation is a conversion of norethisterone acetate to ethinyl oestradiol similar to that reported for norethindrone acetate (28).

The increased sulfonation of both LH and FSH found during the luteal phase of the cycle was suggested to be an effect of progesterone on the enzymes in the sulfonation pathway (2). This hypothesis is further supported in the present study by the increased number of sulfonated residues per LH and FSH molecule during the progestogen therapy.

Women with raised androgen levels, as in the polycystic ovarian syndrome (PCOS), had decreased sulfonation and increased sialylation of LH and FSH when compared with healthy women in the follicular phase (2). These effects of androgens on the glycosylation were thus opposite to those found for the progestogen NETA in the present study.

There is a general agreement that the oligosaccharide heterogeneity on the gonadotrophin molecules has some kind of physiological significance in the human (29–31). In this study, the oligosaccharide variation most likely played an important physiological role when the serum levels of the gonadotrophins decreased during the oestradiol and NETA treatments to mean levels of 6.1% for LH and 3.9% for FSH compared with the non-treated group. The serum level is determined by the secretion rate and the metabolic clearance rate of the gonadotrophin isoforms. More sulfonated residues and fewer sialic acid residues per molecule were associated (*P* < 0.0001) with lower serum levels of the gonadotrophins. More sulfonated and less sialylated gonadotrophins disappear faster from the human circulation (16,17). It seems likely that the increased sulfonation and decreased sialylation of the gonadotrophins substantially contributed to the decreased serum levels during both the oestradiol and the progestogen therapies.

Serum LH isoforms with two or more sulfonated GalNAc residues disappear considerably faster from the circulation than those with zero or one (16,17). This was thought to be due to a rapid removal from the circulation by a human hepatic receptor specific for SO_3_-4GalNAcβ1,4GlcNAcβ1,2Manα structure similar to that found in rodents (32,33). The frequency of LH isoforms with two to four SO_3_-GalNAc residues in the E_2_-implant group was 31.6%, a figure close to that (32%) in younger women at follicular phase (2). During the NETA therapy the frequency increased after 2 and 4 weeks to 56% and 65%, respectively. These values are considerably higher than the 37% reported for the luteal phase of the cycle (2), and the difference may be explained by the progestogen preparation, the dose, and/or the long E_2_ implant treatment of the post-menopausal women. The high frequency of more sulfonated residues is expected to lead to a rapid disappearance of these LH isoforms from the circulation and explain the very low LH serum levels during the NETA treatment in this and in the previous report (11).

The sialic acid residues constitute 96.2% of the negatively charged residues on the oligosaccharides on FSH extracted from human pituitaries taken at autopsy (3). The corresponding mean percentage of sialic acid residues on serum FSH from men and post-menopausal women was 97.2% (2). As the degree of sulfonation of FSH is very low, large changes in charge to less anionic (acidic) FSH isoforms during oestrogen treatment of both men and women have been interpreted as decreases in the sialic acid contents (5–15). We can confirm this interpretation with the results of the present study. On the other hand, a change in charge to more anionic FSH isoforms should be interpreted with caution as it could be due to an increase in sulfonated and/or sialic acid residues.

In this study we have examined the effect of oestradiol and of a progestogen, NETA, on the sialylation and the sulfonation of LH and FSH in E_2_-treated post-menopausal women. The report indicates that the primary effect of the E_2_ treatment was a decrease in the sialylation and, due to competition for the same substrate, secondarily and consequentially a minor increase in the sulfonation of FSH and LH. The effect of 2 weeks of treatment with NETA was primarily an activation of the sulfonation pathway during the synthesis of LH and FSH and, consequentially, also a minor decrease in the sialylation pathway of LH.

## References

[1] Wide L (1985). Median charge and charge heterogeneity of human pituitary FSH, LH and TSH. II. Relationship to sex and age. Acta Endocrinol (Copenh).

[2] Wide L, Naessén T, Sundström-Poroma I, Eriksson K (2007). Sulfonation and sialylation of gonadotropins in women during the menstrual cycle, after menopause, and with polycystic ovarian syndrome and in men. J Clin Endocrinol Metab.

[3] Green ED, Baenziger JU (1988). Asparagine-linked oligosaccharides on lutropin, follitropin, and thyrotropin. II. Distributions of sulfated and sialylated oligosaccharides on bovine, ovine, and human pituitary glycoprotein hormones. J Biol Chem.

[4] Wilson CA, Leigh AJ, Chapman AJ (1990). Gonadotrophin glycosylation and function. J Endocrinol.

[5] Wide L (1982). Male and female forms of human follicle-stimulating hormone in serum. J Clin Endocrinol Metab.

[6] Padmanabhan V, Lang LL, Sonstein J, Kelch RP, Beitins IZ (1988). Modulation of serum follicle-stimulating hormone bioactivity and isoform distribution by estrogenic steroids in normal women and in gonadal dysgenesis. J Clin Endocrinol Metab.

[7] Matikainen T, Haavisto A-M, Permi J, de Kretser D, Huhtaniemi I (1994). Effects of oestrogen treatment on serum gonadotrophin bioactivity, immunoreactivity and isohormone distribution, and on immunoreactive inhibin levels, in prostatic cancer patients. Clin Endocrinol.

[8] Wide L, Naessén T (1994). 17β-Oestradiol counteracts the formation of the more acidic isoforms of follicle-stimulating hormone and luteinizing hormone after menopause. Clin Endocrinol (Oxf).

[9] Wide L, Naessén T, Phillips DJ (1995). Effect of chronic daily oral administration of 17β-oestradiol and norethisterone on the isoforms of serum gonadotropins in post-menopausal women. Clin Endocrinol (Oxf).

[10] Ulloa-Aguirre A, Midgley R, Beitins IZ, Padmanabhan V (1995). Follicle-stimulating isohormones: characterization and physiological relevance. Endocrine Rev.

[11] Wide L, Naessén T, Eriksson K, Rune C (1996). Time-related effects of a progestogen on the isoforms of the serum gonadotropins in the 17β-oestradiol treated post-menopausal women. Clin Endocrinol (Oxf).

[12] Anobile CJ, Talbot JA, McCann SJ, Padmanabhan V, Robertson WR (1998). Glycoform composition of serum gonadotrophins through the normal menstrual cycle and in the post-menopausal state. Mol Hum Reprod.

[13] Padmanabhan V, Christman GM, Randolph JF, Kelch RP, Marshall JC, Beitins IZ (2001). Dynamics of bioactive follicle-stimulating hormone secretion in women with polycystic ovary syndrome: effects of estradiol and progesterone. Fertil Steril.

[14] Velasquez EV, Creus S, Trigo RV, Cigorraga SB, Pellizzari EH, Croxatto HB (2006). Pituitary-ovarian axis during lactational amenorrhoea. II. Longitudinal assessment of serum FSH polymorphism before and after recovery of menstrual cycles. Hum Reprod.

[15] Hernandez-Valencia M, Zarate A, Sandoval A, Ruiz M, Timossi C, Amato D (2007). Conjugated estrogens and tibolone modify the gonadotrophin glycosylation pattern in postmenopausal women. Gynecol Obstet Invest.

[16] Wide L, Eriksson K, Sluss PM, Hall JE (2009). Serum half-life of pituitary gonadotropins is decreased by sulfonation and increased by sialylation in women. J Clin Endocrinol Metab.

[17] Wide L, Eriksson K, Sluss PM, Hall JE (2010). The common genetic variant of luteinizing hormone has a longer serum half-life than the wild type in heterozygous women. J Clin Endocrinol Metab.

[18] Haavisto A-M, Pettersson K, Bergendahl M, Virkamäki A, Huhtaniemi I (1995). Occurrence and biological properties of a common genetic variant of luteinizing hormone. J Clin Endocrinol Metab.

[19] Nilsson C, Jiang M, Pettersson K, Iitiä A, Mäkelä M, Simonsen H (1998). Determination of a common genetic variant of luteinizing hormone using DNA hybridization and immunoassays. Clin Endocrinol (Oxf).

[20] Phillips DJ, Albertsson-Wikland K, Eriksson K, Wide L (1997). Changes in the isoforms of luteinizing hormone and follicle-stimulating hormone during puberty in normal children. J Clin Endocrinol Metab.

[21] Wide L (1985). Median charge and charge heterogeneity of human pituitary FSH, LH and TSH. I. Zone electrophoresis in agarose suspension. Acta Endocrinol (Copenh).

[22] Baenziger JU (1994). Protein-specific glycosyltransferases: how and why they do it!. FASEB J.

[23] Smith PL, Baenziger JU (1992). Molecular basis of recognition by the glycoprotein hormone-specific N-acetylgalactosamine-transferase. Proc Natl Acad Sci U S A.

[24] Mengeling BJ, Manzella SM, Baenziger JU (1995). A cluster of basic aminoacids within an α-helix is essential for α-subunit recognition by the glycoprotein hormone N-acetylgalactosaminyltransferase. Proc Natl Acad Sci U S A.

[25] Dharmesh SM, Baenziger JU (1993). Estrogen modulates expression of the glycosyltransferases that synthesize sulphated oligosaccharides on lutropin. Proc Natl Acad Sci U S A.

[26] Damián-Matsumura P, Zaga V, Maldonado A, Sánchez-Hernández C, Timossi C, Ulloa-Aguirre A (1999). Oestrogens regulate pituitary α2,3-sialyltransferase messenger ribonucleic acid levels in the female rat. J Mol Endocrinol.

[27] Fink B, Svenstrup B, Bennet B, Micic S, Moller S (1988). Effect of oestrogen and progestogen on gonadotrophins and sex hormones in oophorectomized women. Eur J Obstet Gynecol Reprod Biol.

[28] Chu C, Zhang X, Gentzschein E, Stanczyk FZ, Lobo RA (2007). Formation of ethinyl estradiol in women during treatment with norethindrone acetate. J Clin Endocrinol Metab.

[29] Ulloa-Aguirre A, Timossi C, Méndez JP (2001). Is there any physiological role for gonadotrophin oligosaccharide heterogeneity in humans? I. Gonadotrophins are synthesized and released in multiple molecular forms. A matter of fact. Hum Reprod.

[30] Dias JA (2001). Is there any physiological role for gonadotrophin oligosaccharide heterogeneity in humans? II. A biochemical point of view. Hum Reprod.

[31] Bergendahl M, Veldhuis JD (2001). Is there a physiological role for gonadotrophin oligosaccharide heterogeneity in humans? III. Luteinizing hormone heterogeneity: a medical physiologist's perspective. Hum Reprod.

[32] Fiete D, Srivastava V, Hindsgaul O, Baenziger JU (1991). A hepatic reticuloendothelial cell receptor specific for SO4-4GalNAcβ1,4GlcNAcβ1,2Manα that mediates rapid clearance of lutropin. Cell.

[33] Roseman DS, Baenziger JU (2000). Molecular basis of lutropin recognition by the mannose/GalNAc-4-SO4 receptor. Proc Natl Adac Sci U S A.

